# Pediatric Prolonged-Release Melatonin for Sleep in Children with Autism Spectrum Disorder: Impact on Child Behavior and Caregiver’s Quality of Life

**DOI:** 10.1007/s10803-019-04046-5

**Published:** 2019-05-11

**Authors:** Carmen M. Schroder, Beth A. Malow, Athanasios Maras, Raun D. Melmed, Robert L. Findling, John Breddy, Tali Nir, Shiri Shahmoon, Nava Zisapel, Paul Gringras

**Affiliations:** 1 Department of Child and Adolescent Psychiatry & CIRCSom, Strasbourg University Hospital, Strasbourg, France; 2 CNRS UPR 3212, Institute of Cellular and Integrative Neurosciences, Strasbourg, France; 3 Sleep Division, Department of Neurology, Vanderbilt University Medical Center, Nashville, TN USA; 4 Yulius Academy, Yulius Mental Health Organization, Dordrecht, Delft, The Netherlands; 5 Southwest Autism Research and Resource Center, Scottsdale, Phoenix, AZ USA; 6 Department of Psychiatry and Behavioral Sciences, Kennedy Krieger Institute/Johns Hopkins University, Baltimore, MD USA; 7 Pharmastat Consulting Ltd, Canterbury, UK; 8 Neurim Pharmaceuticals Ltd, Tel Aviv, Israel; 9 Children’s Sleep Medicine, Evelina London Children’s Hospital, Guy’s and St Thomas’, London, UK

**Keywords:** Prolonged-release melatonin, Children (pediatric), Sleep, Autism, Behavior

## Abstract

A randomized, 13-weeks, placebo-controlled double-blind study in 125 subjects aged 2–17.5 years with Autism Spectrum Disorder or Smith-Magenis syndrome and insomnia demonstrated efficacy and safety of easily-swallowed prolonged-release melatonin mini-tablets (PedPRM; 2–5 mg) in improving sleep duration and onset. Treatment effects on child behavior and caregiver’s quality of life were evaluated. PedPRM treatment resulted in significant improvement in externalizing but not internalizing behavior (Strengths and Difficulties questionnaire; SDQ) compared to placebo (p = 0.021) with clinically-relevant improvements in 53.7% of PedPRM-treated versus 27.6% of placebo-treated subjects (p = 0.008). Caregivers’ quality of life also improved with PedPRM versus placebo (p = 0.010) and correlated with the change in total SDQ (p = 0.0005). PedPRM alleviates insomnia-related difficulties, particularly externalizing behavior in the children, subsequently improving caregivers’ quality of life.

## Introduction

Insomnia disorder is a complaint on recurrent poor sleep quality or quantity characterized by difficulty initiating and/or staying asleep, and/or nonrestorative sleep causing distress or impairment in important areas of functioning (APA 2013). Insomnia disproportionately affects children with Autism Spectrum Disorder (ASD) and distinct Neurogenetic Disorders (NGD, e.g. Smith-Magenis syndrome (SMS), Angelman syndrome, Rett syndrome, Bourneville syndrome (tuberous sclerosis), Williams Syndrome) compared to neurotypical (typically developing; TD) children (Mindell et al. 2006; Sivertsen et al. 2012; Reilly et al. 2015). The most common complaints reported are difficulties falling asleep (~ 40%) and difficulties maintaining sleep (~ 35%) (Taira et al. 1998; Krakowiak et al. 2008). These difficulties persist from infancy to adolescence (Humphreys et al. 2014). Insomnia may precede ASD diagnosis; infants with more sleep challenges by 12 months, especially those waking more often during the night, showed an increased number of early ASD symptoms a year later (Nguyen et al. 2018).

Previous studies in TD children have shown that shortened sleep duration in early childhood is associated with externalizing behaviors (behaviors that are directed toward the external environment such as hyperactivity-impulsivity, or aggression), and lower cognitive performance on neurodevelopmental tests (Lavigne et al. 1999; Touchette et al. 2007; Reid et al. 2009). In addition, many studies have indicated a potential link between insufficient sleep and attention deficit hyperactivity disorder (ADHD) in children (Lavigne et al. 1999). Similarly, children with ASD and poor sleep demonstrate significantly higher daytime behavioral problems, including irritability, externalizing behavior (specifically hyperactivity and aggression), and internalizing behaviors (behaviors lashing out at the self, such as social withdrawal, anxiety depression)- compared to those who sleep well (Goldman et al. 2011; Henderson et al. 2011; Johnson et al. 2018).

Attention-deficit/hyperactivity disorder (ADHD) is one of the most common co-occurring conditions seen in individuals with neurodevelopmental difficulties, specifically ASD or NGD (Murray 2010; Matson et al. 2013). Sleep problem severity in ASD is similar across ADHD and non-ADHD subgroups (Taira et al. 1998; Krakowiak et al. 2008). If there is a causal link between poor sleep and exacerbation of behavioral abnormalities, alleviation of sleep problems in children with ASD/NGD, in particular those with co-occurring ADHD symptoms, should have a positive effect on these behavioral attributes.

The severity of sleep problems is of particular concern in light of the increased burden and stress experienced in parenting a child with ASD along with the potentially adverse impact of sleep disturbances and insufficient sleep duration on a child’s daytime behavior and cognitive functioning (Patzold et al. 1998; Honomichl et al. 2002; Allik et al. 2006; Doo and Wing 2006). Current practice holds that parent directed behavioral sleep interventions including sleep hygiene should be the first line approach in treating pediatric sleep problems. Children with ASD have shown a 25% response rate to behavioral interventions (Gringras et al. 2012). Pharmacotherapy is often initiated when sleep interventions fail despite there being no approved drugs for the treatment of insomnia in the pediatric population (Johnson and Malow 2008). There is a strong consensus among researchers that exogenous melatonin is beneficial for treating chronic sleep–wake cycle disorders in children with neurodevelopmental and neuropsychiatric disorders (Rossignol 2009; Cuomo et al. 2017).

In particular, immediate release formulations of melatonin were effective in facilitating sleep onset but less so in prolonging the total amount of sleep. As melatonin has a very short half-life (40 min), a prolonged-release melatonin (PRM) formulation designed to mimic the endogenous profile by releasing melatonin throughout the night, may help improve both sleep initiation and maintenance (De Leersnyder 2006; De Leersnyder et al. 2011).

Paediatric Prolonged-Release Melatonin (Slenyto®, Neurim Pharmaceuticals; PedPRM) is a novel age appropriate formulation developed for sleep disorders in children with neurodevelopmental disabilities that have difficulties swallowing. PedPRM is an oral solid dosage form of prolonged-release melatonin mini-tablets (3 mm in diameter), to be swallowed as a whole and has been designed to gradually release melatonin, mimicking the physiological secretion profile of melatonin to produce sustained plasma levels of melatonin for up to 8–10 h.

We have recently reported on a 13 week, randomized, double-blind, placebo-controlled, parallel group, multi-center (EU and USA) study on the effects of Ped-PRM, in children and adolescents (ages 2–17.5 years old; N = 125) with ASD or SMS (with or without co-occurring ADHD), who had not shown improvement after standard sleep hygiene intervention (Gringras et al. 2017). Study results indicated that PedPRM (2/5 mg) was efficacious and safe compared to placebo for treatment of insomnia in children with ASD or SMS with clinically meaningful improvements shown in total sleep time (TST), duration of uninterrupted sleep (longest sleep episode) and sleep latency (SL) and without causing earlier wake-up time (Gringras et al. 2017). Subjects slept on average 57.5 min longer at night with PedPRM compared to 9.14 min with placebo (adjusted mean treatment difference PedPRM-placebo TST = − 32.43 min; *p *= 0.034). Sleep Latency (SL) decreased by 39.6 min on average with PedPRM and 12.5 min with placebo (adjusted mean treatment difference SL = − 25.30 min; *p *= 0.011) without causing earlier wake-up time. Rate of participants attaining clinically meaningful responses in TST and/or SL (increase ≥ 45 min in TST and/or decrease ≥ 15 min in SL) was significantly higher with PedPRM than placebo (68.9% vs. 39.3% respectively; *p *= 0.001) (Gringras et al. 2017).

The effects of PedPRM intervention compared with placebo on child behavior as well as caregiver’s well-being outcomes in children with ASD and insomnia were studied.

## Methods

### Study Design and Participants

A randomized, double-blind, placebo-controlled trial was conducted at 14 centers in the United States (US) and 10 centers in Europe. The study design and participants were as recently described (Gringras et al. 2017). The trial complied with the principles of the Declaration of Helsinki (1989) and standards of good clinical practices. All participants and parents/legal guardians provided written, signed informed assent and consent, respectively, prior to participation, under procedures and local regulations of each country. ClinicalTrials.gov Identifier: NCT01906866

### Procedures

Children (ages 2–17.5 years) with: (1) physician-diagnosed ASD according to ICD-10 or DSM-IV/DSM-5 criteria, or NGD [while the protocol allowed SMS, Angelman syndrome, and Bourneville’s disease (tuberous sclerosis), eventually only SMS patients participated] along with (2) chronic sleep disturbances (minimum 3 months of impaired sleep defined as ≤ 6 h of continuous sleep AND/OR ≥ 0.5 h sleep latency from light-off in 3 of 5 nights based on parent report and patient medical history) were included.

Children without documented history of sleep hygiene intervention at screening, underwent 4 weeks of booklet-assisted basic parent-led sleep behavioral interventions based on a previously studied and standardized sleep behavior treatment (Montgomery et al. 2004). This period ensured that children whose sleep disorder was amenable to treatment with non-pharmacological intervention were not randomized, along with serving as an opportunity for wash-out from any hypnotics.

Exclusion criteria included other sleep disorders according to medical history, use of prohibited medication or melatonin within 2 weeks prior to screening, allergy to melatonin or lactose (an excipient of the mini-tablet) or unresponsive to previous of PRM (Slenyto®, Neurim Pharmaceuticals) therapy and participation in a clinical trial within the last 3 months prior to the study. Females not using contraceptives that were sexually active, pregnant and/or breastfeeding females were excluded (Gringras et al. 2017).

Eligible children entered a 2-week single-blind placebo run-in period after which, if their impaired sleep continued [defined as ≤ 6 h of continuous sleep AND/OR ≥ 0.5 h sleep latency from light-off in 3 out of 5 nights in the last two weeks, based on parent-reported Sleep and Nap Diary (SND)] (Carney et al. 2012), they were randomly assigned (1:1), via permuted block randomization (using an eCRF system), to receive either PedPRM or placebo for the 13-week double-blind treatment period. Placebo mini-tablets were identical in appearance and formulation to PedPRM mini-tablets but without melatonin. The starting dose was 2 mg PedPRM (2 × 1 mg) or matched placebo (2 × 0 mg) mini-tablets once-daily 30–60 min before habitual bedtime and after or with food. After 3 weeks of double-blind treatment, sleep variables were assessed. If the patient did not improve from baseline by at least 1 h as measured by shortening of SL and/or increase in TST, the dose was escalated to 5 mg PedPRM or a matched number of placebo mini-tablets. Optional decrease in dose was also allowed at all times during the study, based on evaluation of excess drowsiness, behavioral changes or ceasing to respond to study drug according to the double-blind phase dose. Children then continued in the double-blind period on the same dose of PedPRM or matched placebo mini-tablets for the remaining 10 weeks, with an efficacy assessment performed at the end of the 13 weeks double-blind treatment period.

All completers of the double blind phase then received open label PedPRM (2/5 mg) according to the final dose in the double-blind phase. Accordingly, in the placebo arm, those who received 2 mini-tablets of placebo received 2 mg PedPRM and those who received 5 placebo mini-tablets received 5 mg PedPRM in the open-label phase. Children then continued in a 91-week open-label follow-up treatment period. After the first 13 weeks of open label treatment there was a second optional dose optimization (2, 5 or 10 mg/day) for those who did not improve by 60 min or more in TST, SL or both from baseline. Subjects then continued open-label on 2, 5 or 10 mg of PedPRM for the remaining period, with efficacy assessment after 26, 39 and 91 weeks of open-label follow-up. The PedPRM treatment period was followed by 2 week single-blind placebo period (withdrawal phase), with the overall study period being 2.2 years.

### Child Behavior and Caregiver’s Outcomes

Child sleep and caregivers’ outcomes were assessed throughout the trial. In addition, child behavior was assessed in the double blind phase. Child behavior outcomes were evaluated using the SDQ. Caregiver’s well-being outcomes were evaluated using the caregiver’s World Health Organization Well-Being Index (WHO-5) the Pittsburgh Sleep Quality Index (PSQI) and The Epworth Sleepiness Scale (ESS).

The SDQ is a validated behavioral screening tool comprising 25 items on 5 psychological attributes including hyperactivity/inattention (5 items), conduct problems (5 items), peer relationship problems (5 items), emotional symptoms (5 items), prosocial behavior (5 items) along with an impact supplement (Goodman 1999). Validation studies of the SDQ showed three independent domains pertaining to externalizing behavior [sum of hyperactivity/inattention (5 items) and conduct problems (5 items)], internalizing behavior [sum of peer relationship problems (5 items) and emotional symptoms (5 items)] and prosocial behavior (5 items) (Goodman et al. 2010). The externalizing and internalizing behavior scores showed good convergent and discriminant validity across informants and with respect to clinical observation and thus were determined to be more appropriate outcome variables for clinical studies (Goodman et al. 2010). The sum of the externalizing and internalizing attributes (Hyperactivity, Conduct, Peer relationship and Emotion subscales) are aggregated to form a Total Difficulties Score (total SDQ); a higher score indicates poorer behavioral adjustment. The third factor, prosocial behavior is excluded from the total SDQ. The total SDQ score has been found to be a psychometrically sound measure of overall child mental health problem (Goodman 1999). A change of any 1 unit across the SDQ range is considered clinically relevant as the odds of mental disorders increased at a constant rate across the range (odds ratios between 1.14 and 1.28 per one-point increase in SDQ score (Goodman & Goodman 2009)). A total SDQ score ≥ 20, a score of ≥ 7 on the SDQ hyperactivity/inattention attribute, a peer relationship score ≥ 6, an emotional score ≥ 7 and a conduct score ≥ 5, are considered abnormal using the published cutoff scores (available from http://www.sdqinfo.com/py/sdqinfo/c0.py) (Goodman 1997).

The WHO-5 Wellbeing index (Bech et al. 2003) is a 25 point index that covers positive mood, vitality, and general interests. Effect size of 0.4 and over is considered clinically relevant (Bech et al. 2007).

The Pittsburgh Sleep Quality Index (PSQI) comprises nine main questions relating to the patient’s usual sleep habits during the previous 2 weeks. It addresses possible reasons for trouble in sleeping as well as daytime behavior. The caregiver is asked to give the most accurate reply for the majority of his/her own days and nights during this period. An algorithm is used to calculate seven component scores and these are added to give a global PSQI score. The PSQI has been recommended as an essential measure for global sleep and insomnia symptoms in recent expert consensus recommendations for a standard set of research assessments in insomnia (Buysse et al. 2006).

The Epworth Sleepiness Scale (ESS) is a self-administered questionnaire with 8 questions. Caregivers were asked to rate, on a 4-point scale (0–3), their usual chances of dozing off or falling asleep while engaged in eight different activities. The higher the ESS score, the higher that person’s average sleep propensity in daily life, or their ‘daytime sleepiness’ (Johns 1991).

Safety was monitored throughout the study, using standard clinical trials methods and definitions [Treatment-Emergent Signs and Symptoms (TESS), Adverse Events (AEs), vital signs and physical examination] and epilepsy events. Standard age appropriate methods [Tanner scales for pubertal development, Body Mass Index (BMI) Z scores for growth], were used to assess the children’s development and health status.

### Statistical Methods

Efficacy analyses are presented for the Full Analysis Subset (FAS), comprising all patients in the Safety Analysis Set (who take at least one dose of study medication) who satisfy all major entry criteria and who have efficacy data for the primary variable at baseline and at least one during the double-blind phase. Variables were analyzed using a mixed-effects model for repeated-measures (MMRM) that included fixed effects for visit, mean baseline value, randomized treatment and mean baseline value and randomized treatment both nested within visit. Visit-to-visit repeated measures assumed unstructured covariance structure. Within-participant changes from baseline were analyzed using paired t-tests. These analyses were performed also for subpopulations of the cohort with abnormal behavior scores at baseline (hyperactivity/inattention, conduct, peer relationship, emotional or total difficulties).

To evaluate whether and to what extent the improvement in sleep could explain the improvements in behavior or in caregivers’ quality of life, we analyzed the correlation between the change in total SDQ and the main sleep variables in the study, namely TST, sleep latency and duration of uninterrupted sleep (longest sleep episode) in the total sample (PedPRM and placebo groups together). Similarly, the correlations between the change in caregiver’ quality of life and the changes in the mean sleep variables and in total SDQ were analyzed (Spearman’s rank correlation).

## Results

### Participants

**Fig. 1:**
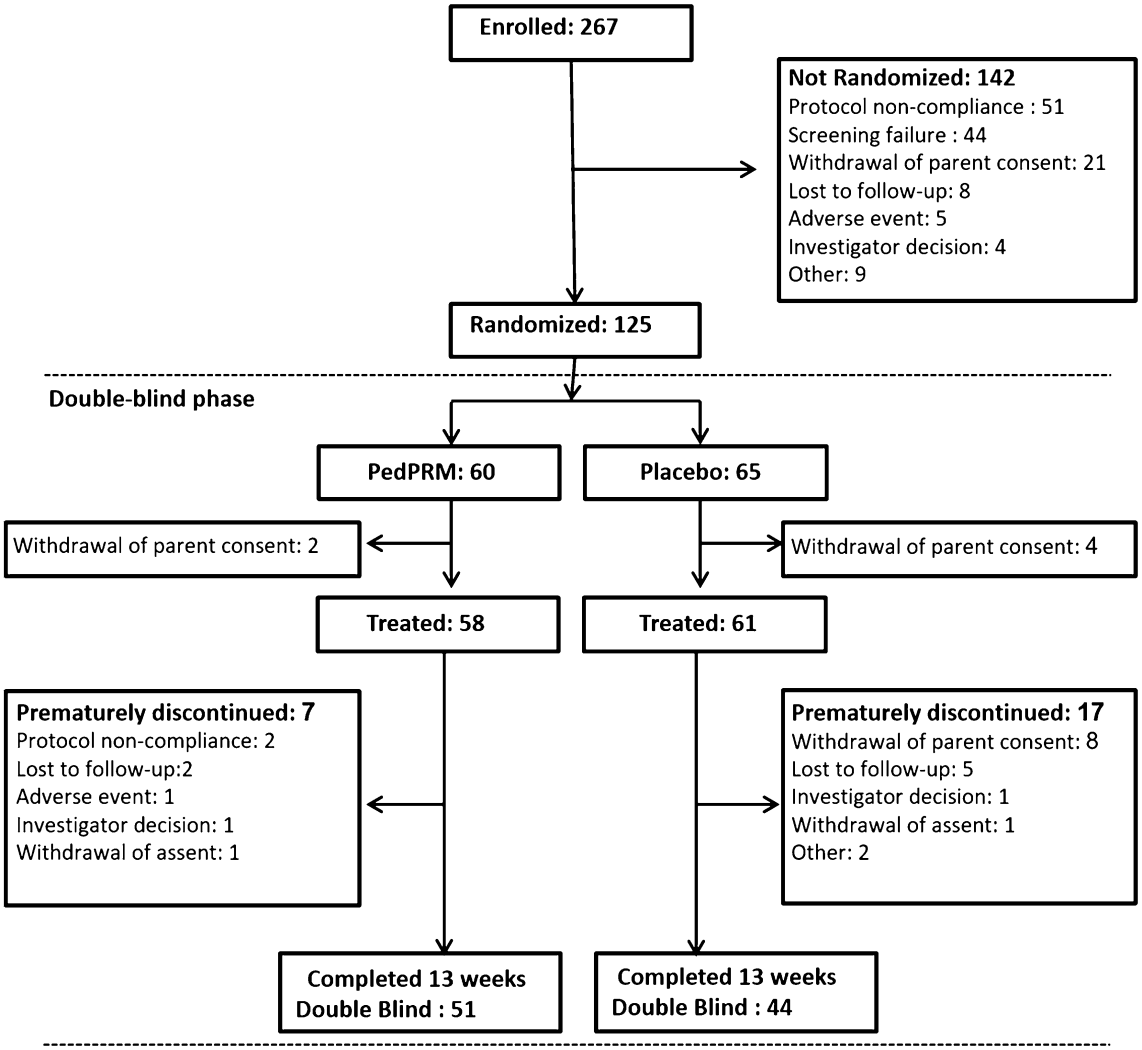
CONSORT diagram of the randomization double blind period of the study participants

Patient population was detailed elsewhere (Gringras et al. 2017). In brief, 125 subjects were randomized in the double-blind phase, 119 were treated (58 patients in the PedPRM group and 61 patients in the placebo group) and 95 patients completed the double-blind phase (Fig. 1). There were no notable differences between the randomization groups regarding demography, baseline disease characteristics and medical history (Table 1). Most randomized patients had a diagnosis of ASD (96.8%) at screening; the remaining patients (3.2%) had Smith-Magenis Syndrome (SMS) with associated neurodevelopmental disabilities. Of the randomized participants, 36 (28.8%; 16 in the PedPRM and 20 in the placebo group) had co-occurring ADHD according to their medical history records. All patients reported impaired sleep and most (83.2%) had undergone previous sleep hygiene training. Mean treatment compliance was close to 100% in both treatment groups throughout the study.

**Table 1:** Demographic and baseline characteristics of randomized groups

	Circadin®	Placebo	Overall
	(N = 60)	(N = 65)	(N = 125)
Age, years			
Mean ± SD	9.0 ± 4.08	8.4 ± 4.24	8.7 ± 4.15
Range	2, 17	2, 17	2, 17
Sex, n (%)			
Male	45 (75.0%)	47 (72.3%)	92 (73.6%)
Female	15 (25.0%)	18 (27.7%)	33 (26.4%)
Ethnicity, n (%)			
Not Hispanic or Latino	40 (66.7%)	49 (75.4%)	89 (71.2%)
Hispanic or Latino	12 (20.0%)	7 (10.8%)	19 (15.2%)
Other	8 (13.3%)	8 (12.3%)	16 (12.8%)
Unknown	0	1 (1.5%)	1 (0.8%)
Race, n (%)			
White	57 (95.0%)	55 (84.6%)	112 (89.6%)
Black or African American	1 (1.7%)	8 (12.3%)	9 (7.2%)
Other	3 (5.0%)	3 (4.6%)	6 (4.8%)
Asian	0	2 (3.1%)	2 (1.6%)
Height, cm			
Mean ± SD	133.4 ± 24.17	130.4 ± 27.20	131.8 ± 25.73
Range	89, 180	79, 197	79, 197
Weight, kg			
Mean ± SD	37.86 ± 21.495	35.22 ± 23.249	36.49 ± 22.374
Range	11.7, 90.1	9.8, 129.9	9.8, 129.9
BMI, kg/m^2^			
Mean ± SD	19.50 ± 4.899	18.79 ± 4.901	19.13 ± 4.893
Range	12.7, 32.8	12.3, 35.3	12.3, 35.3
Disease characteristics			
ASD	58 (96.7%)	63 (96.9%)	121 (96.8%)
SMS	2 (3.3%)	2 (3.1%)	4 (3.2%)
ADHD	16 (26.7%)	20 (30.8%)	36 (28.8%)
Epilepsy	10 (16.7%)	6 (9.2%)	16 (12.8%)
Impaired sleep	60 (100%)	65 (100%)	125 (100%)
Medical history			
Agitation	42 (70.0%)	46 (70.8%)	88 (70.4%)
Mood swings	43 (71.7%)	40 (61.5%)	83 (66.4%)
Somnolence	28 (46.7%)	35 (53.8%)	63 (50.4%)
Fatigue	35 (58.3%)	36 (55.4%)	71 (56.8%)
Behavioral intervention	51 (85.0%)	53 (81.5%)	104 (83.2%)

After 3 weeks of double-blind treatment (starting at study week 3), 41% (23 of 56) of the children in the PedPRM treatment group improved their sleep on the 2 mg dose versus 20% (12 of 61) in the placebo group. The remaining subjects 59% (33 of 56) in the PedPRM group and 80% (49 of 61) in the placebo group were escalated (at week 5) to the higher dose of 5 mg PedPRM or the placebo equivalent. We did not find any differences between children improving on 2 versus 5 mg of PedPRM.

### Child Behavior Outcomes

The effects of PedPRM and placebo treatments on the subject’s behavior as assessed by the SDQ are depicted in Table 2. PedPRM treatment (13 weeks) resulted in statistically significant improvements in mean SDQ-assessed externalizing behavior and tendencies to improve in total SDQ and impact score compared to placebo (Table 2). Attributes related to internalizing behaviors (peer relationship and emotional behavior) mean scores were not significantly affected in the total population (Table 2).

**Table 2:** Behavior (SDQ) after 13 weeks of double blind treatment

Variable	Group	Adjusted treatment means (SE) [95% CI]	Treatment difference (SE)	95% CI	p value*
SDQ					
Externalizing behavior	PedPRM	− 0.70 (0.244) [− 1.19; − 0.22]	− 0.83 (0.355)	− 1.54, − 0.13	0.021
	Placebo	0.13 (0.258) [− 0.38; 0.64]			
Total score	PedPRM	− 0.84 (0.387) [− 1.61, − 0.07]	− 1.01 (0.563)	− 2.12, 0.11	0.077
	Placebo	0.17 (0.409) [− 0.64, 0.98]			
Impact score	PedPRM	− 0.57 (0.283) [− 1.13, − 0.01]	− 0.74 (0.411)	− 1.55, 0.08	0.076
	Placebo	0.16 (0.298) [− 0.43, 0.76]			
SDQ items					
Hyperactivity/inattention	PedPRM	− 0.47 (0.200) [− 0.87,− 0.08]	− 0.54 (0.290)	− 1.12, 0.03	0.065
	Placebo	0.07 (0.210) [− 0.35, 0.48]			
Conduct problems	PedPRM	− 0.24 (0.138) [− 0.51, 0.04]	− 0.29 (0.199)	− 0.69, 0.11	0.149
	Placebo	0.05 (0.144) [− 0.23, 0.34]			
Peer relationship problems	PedPRM	− 0.02 (0.152) [− 0.32, 0.28]	− 0.05 (0.222)	− 0.49, 0.39	0.811
	Placebo	0.03 (0.161) [− 0.29, 0.35]			
Emotional symptoms	PedPRM	− 0.11 (0.226) [− 0.56, 0.34]	− 0.10 (0.328)	− 0.75, 0.55	0.770
	Placebo	− 0.02 (0.238) [− 0.49, 0.45]			

*MMRM analysis compared to placebo

**Fig. 2:**
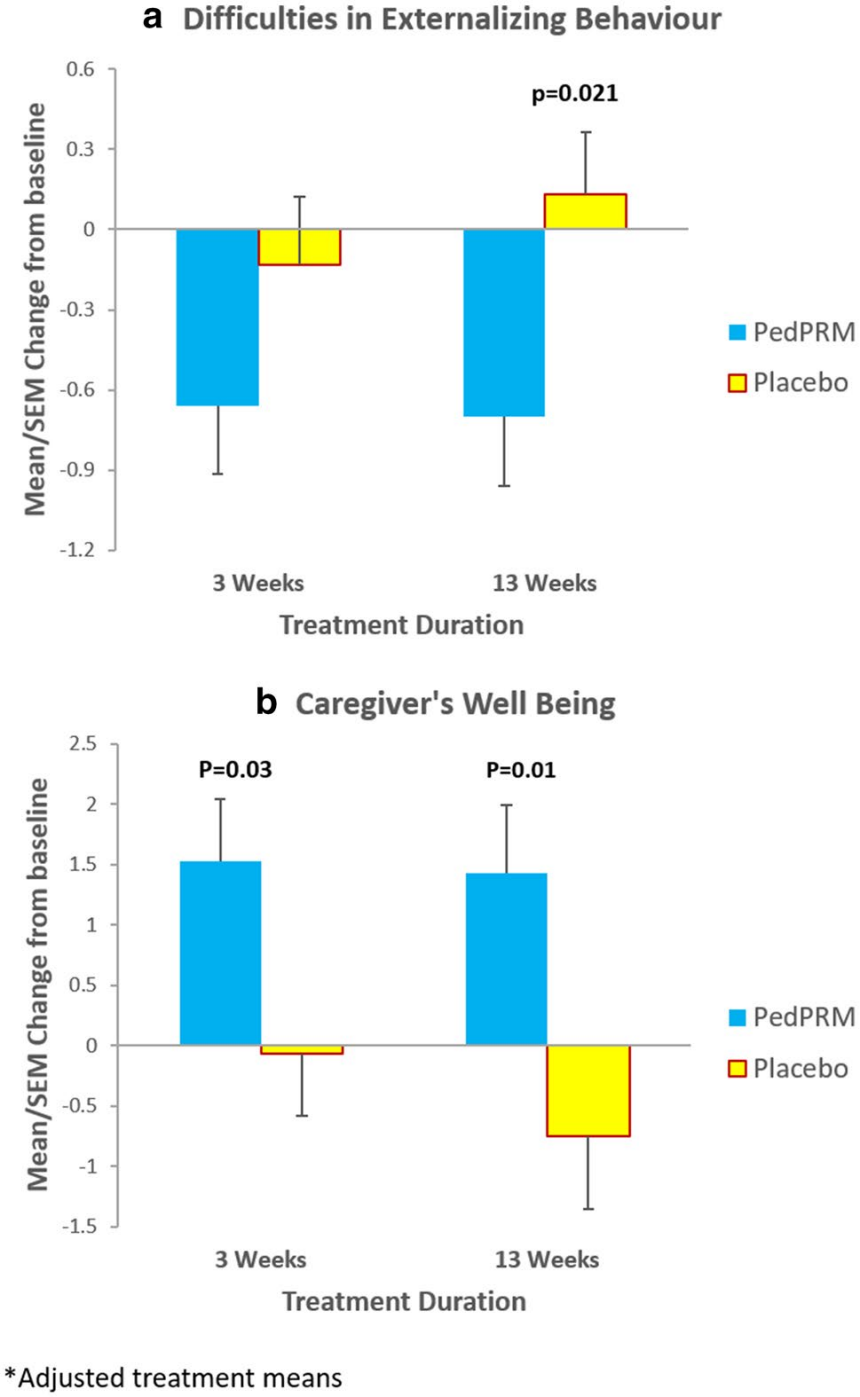
Effects of PedPRM and placebo on Change from baseline (adjusted means (SE)) in: **a** SDQ externalizing behavior during the double-blind period. **b** WHO-5 Caregiver’s well-being and quality of life during the double-blind period

Changes from baseline in mean externalizing behavior scores during the double-blind phase are shown in Fig. 2a. By the end of the 13 weeks, externalizing behavior score improved (decreased) on average − 0.70 units in the PedPRM-treated (N = 54), compared to worsening (increase) of 0.13 units in the placebo-treated group (N = 49) and the adjusted mean difference from placebo in externalizing behavior score in PedPRM-treated subjects was− 0.83 (p = 0.021) (Table 2). Using improvement (i.e. decrease) in externalizing behavior score by 1 unit or more as a criterion of clinical response (Goodman and Goodman 2009), the percentage of responders after 13 weeks of double-blind treatment in the PedPRM group was 53.7% (29 of 54), compared to 27.7% (13 of 47) in the placebo-treated group (Odds ratio 3.0; p = 0.008), providing evidence for the clinical meaningfulness of the treatment effect. The 26% difference in percentage between the groups corresponds to an NNT (number needed to treat) of 3.8.

Total SDQ score (sum of externalizing and internalizing behavior) after 13 weeks of double blind treatment, improved (decreased) significantly in the PedPRM treated group (p < 0.05) but not in the placebo treated group (Table 2). The estimated mean treatment difference between PedPRM and placebo for the change from baseline in total SDQ score was − 1.01 with a trend of benefit in favor of PedPRM (p = 0.077). In the PedPRM group, 26/54 (47%) of subjects had a reduction of 1 or more in total SDQ score compared to 13/47 (27.1%) in the placebo group (p = 0.035). In addition, patients showed a significant improvement in SDQ impact score by the end of the double- blind treatment period in the PedPRM treated group (p < 0.05) but not in the placebo treated group (Table 2). The estimated mean treatment difference between PedPRM and placebo for the change from baseline in SDQ impact score after the 13 weeks of DB treatment was − 0.74 with a trend to benefit, in favor of PedPRM (p = 0.076; Table 2).

On individual attributes, the greatest treatment effect in the total study population was for the hyperactivity/inattention scores. By the end of the 13-week of double-blind treatment, mean SDQ hyperactivity/inattention score improved significantly with PedPRM and worsened with placebo. The estimated mean treatment difference between PedPRM and placebo for the change from baseline in SDQ hyperactivity/inattention score after the 13 weeks of DB treatment was − 0.54 with a trend to benefit, in favor of PedPRM versus placebo (p = 0.065) (Table 2). Conduct problem scores also worsened with placebo and improved with PedPRM but not significantly over placebo.

The study population was quite heterogeneous in behavioral attributes: 73 of 95 (77%) patients completing the double-blind phase with SDQ assessments had a SDQ hyperactivity/inattention score of ≥ 7, 54 (57%) had a total SDQ score ≥ 20, 37 (39%) had a peer relationship score ≥ 6, 24 (25%) had an emotional score ≥ 7 and 15 (17%) had a conduct score ≥ 5 at baseline, all considered abnormal. The effects of the drug and placebo in the subpopulations with abnormal baseline scores in the specific behavioral attributes were thus analyzed independently (Table 3). In the subpopulation of subjects with a score (≥ 7) on the SDQ hyperactivity/inattention attribute, PedPRM treatment (13 weeks) resulted in significant improvement (decrease) in externalizing behavior compared to placebo treatment with a mean treatment difference between PedPRM and placebo of − 0.85 (95% CI − 1.65, − 0.05; p = 0.037). In this subpopulation, the total SDQ also improved significantly in the PedPRM treated group (p = 0.013; Table 3) but not in the placebo treated group with a mean treatment difference over placebo of − 1.46 (95% CI − 2.73, − 0.20; p = 0.024). Furthermore, in the subpopulation with abnormal conduct scores, conduct improved significantly in the PedPRM-treated group but not in the placebo-treated group (Table 3) with a mean treatment difference over placebo of − 1.11(95% CI − 2.05, − 0.17; p = 0.022).

**Table 3:** Effects of 13 weeks PedPRM and placebo treatment on total SDQ and respective behavior scores in subpopulations of subjects with abnormal behavior scores at baseline

Subpopulation	Treatment	N	Total SDQ adjusted treatment means (SE) [95% CI] at 13 weeks	p value*	Respective behavior adjusted treatment means (SE) [95% CI] at 13 weeks	p value*
Abnormal SDQ score ≥ 20	PedPRM	32	− 1.24 (0.496) [− 2.22, − 0.25]	0.012	− 1.24 (0.496) [− 2.22, − 0.25]	0.012
	Placebo	22	− 0.12 (0.553) [− 1.21, 0.98)	ns	− 0.12 (0.553) [− 1.21, 0.98)	
Abnormal-hyperactivity score ≥ 7	PedPRM	42	− 1.06 (0.428) [− 1.91, − 0.21]	0.013	− 0.69 (0.221) [− 1.13, − 0.26]	0.002
	Placebo	31	0.40 (0.487) [− 0.57, 1.37]	ns	− 0.20 (0.248) [− 0.69, 0.29]	ns
Abnormal conduct score ≥ 5	PedPRM	8	− 0.51 (1.026) [− 2.54, 1.53]	0.64	− 0.78 (0.355) [− 1.48, − 0.08]	0.028
	Placebo	7	1.47 (0.922) [− 0.36, 3.30]	ns	0.33 (0.314) [− 0.30, 0.95]	ns
Abnormal peer relationship score ≥ 6	PedPRM	20	− 1.03 (0.598) [− 2.22, 0.16]	0.079	− 0.50 (0.229) [− 0.96, − 0.04]	0.029
	Placebo	17	0.15 (0.706) [− 1.25, 1.55)	ns	− 0.20 (0.255) [− 0.71, 0.30]	ns
Abnormal emotional score ≥ 7	PedPRM	12	− 1.57 (0.803) [− 3.16, 0.03]	0.050	− 1.31 (0.449) [− 2.20, − 0.42]	0.004
	Placebo	12	0.19 (0.819) [− 1.43, 1.82]	ns	− 1.32 (0.460) [− 2.24, − 0.41]	0.003

*Paired T-test from the change from baseline

*ns* not significant

In subjects with abnormal scores in total SDQ, and subjects with abnormal scores in internalizing behaviors (peer relationship, and emotional behavior) at baseline, PedPRM treatment resulted in significant improvement in all respective behavioral attributes as compared to baseline (Table 3). Placebo treatment did not induce significant improvements in total SDQ and peer relationship, but did improve emotional behavior scores in the respective subpopulation (Table 3).

MMRM analysis including co-occurring ADHD diagnosis as a factor, showed that the treatment effects on sleep variables (Gringras et al. 2017) as well as improvement in total SDQ did not differ significantly between the subgroups of participants with co-occurring ADHD as compared to those without ADHD diagnosis (adjusted mean difference of − 1.40 (95% CI − 3.45, 0.65) versus − 0.89 (95% CI − 2.25, 0.47).

The improvement (decrease) in total SDQ score correlated modestly but significantly with the improvement (increase) in TST from baseline (Spearman’s rank correlation R − 0.229 p = 0.024) which could be attributed to the improved duration of uninterrupted sleep (Spearman’s rank correlation R − 0.21 p = 0.047) but not with the shortening of sleep latency (Spearman’s rank correlation R 0.09 p = 0.379).

### Caregiver Related Outcomes

The effects of PedPRM and placebo treatments on caregiver related outcomes as assessed by WHO-5, PSQI and ESS are depicted in Table 4. PedPRM treatment (13 weeks) resulted in statistically significant improvements in mean WHO-5 -assessed quality of life and PSQI assessed caregiver’s sleep quality compared to baseline. Caregivers’ sleepiness (ESS) also improved but not significantly. Placebo treatment did not result in improvements in any of these outcomes (Table 4).

**Table 4:** Caregivers responses after 3 and 13 weeks of double blind treatment

Variable	Group	Adjusted treatment means (SE) [95% CI]	Treatment difference (SE)	95% CI	p-value*
3 weeks					
WHO-5	PedPRM	1.53 (0.515) [0.51; 2.55]	1.60 (0.729)	0.16, 3.05	0.030
	Placebo	− 0.07 (0.516) [− 1.09; 0.95]			
PSQI	PedPRM	− 1.09 (0.360) [− 1.80, − 0.38]	− 0.75 (0.510)	− 1.76, 0.26	0.146
	Placebo	− 0.34 (0.361) [− 1.06, 0.37]			
ESS	PedPRM	− 0.84 (0.395) [− 1.62, − 0.06]	− 1.04 (0.561)	− 2.15, 0.07	0.067
	Placebo	0.02 (0.396) [− 0.59, 0.99]			
13 weeks					
WHO-5	PedPRM	1.43 (0.565) [0.31, 2.55]	2.17 (0.831)	0.53, 3.82	0.010
	Placebo	− 0.75 (0.608) [− 1.95, 0.46]			
PSQI	PedPRM	− 1.11 (0.395) [− 1.89, − 0.32]	− 0.81 (0.582)	− 1.97, 0.34	0.166
	Placebo	− 0.29 (0.427) [− 1.14, 0.55]			
ESS	PedPRM	− 0.74 (0.510) [− 1.76, 0.27]	− 1.29 (0.752)	− 2.78, 0.20	0.089
	Placebo	0.55 (0.552) [− 0.55, 1.64]			

*MMRM analysis compared to placebo

Change from baseline in mean caregivers’ quality of life during the double-blind phase is shown in Fig. 2b. As can be seen, there was a significant improvement in caregiver’s quality of life as assessed by the WHO-5 after 13 weeks of double-blind treatment with treatment difference between the PedPRM treated group and the placebo treated group of 2.17 points (p = 0.01) and Cohen’s d effect size (Cohen 1992) of 0.52. Treatment effects were already evident after 3 weeks of treatment (Fig. 2b; Table 4); the treatment difference (PedPRM vs. placebo) after 3 weeks was 1.60 points (p = 0.03) at that time.

**Fig. 3:**
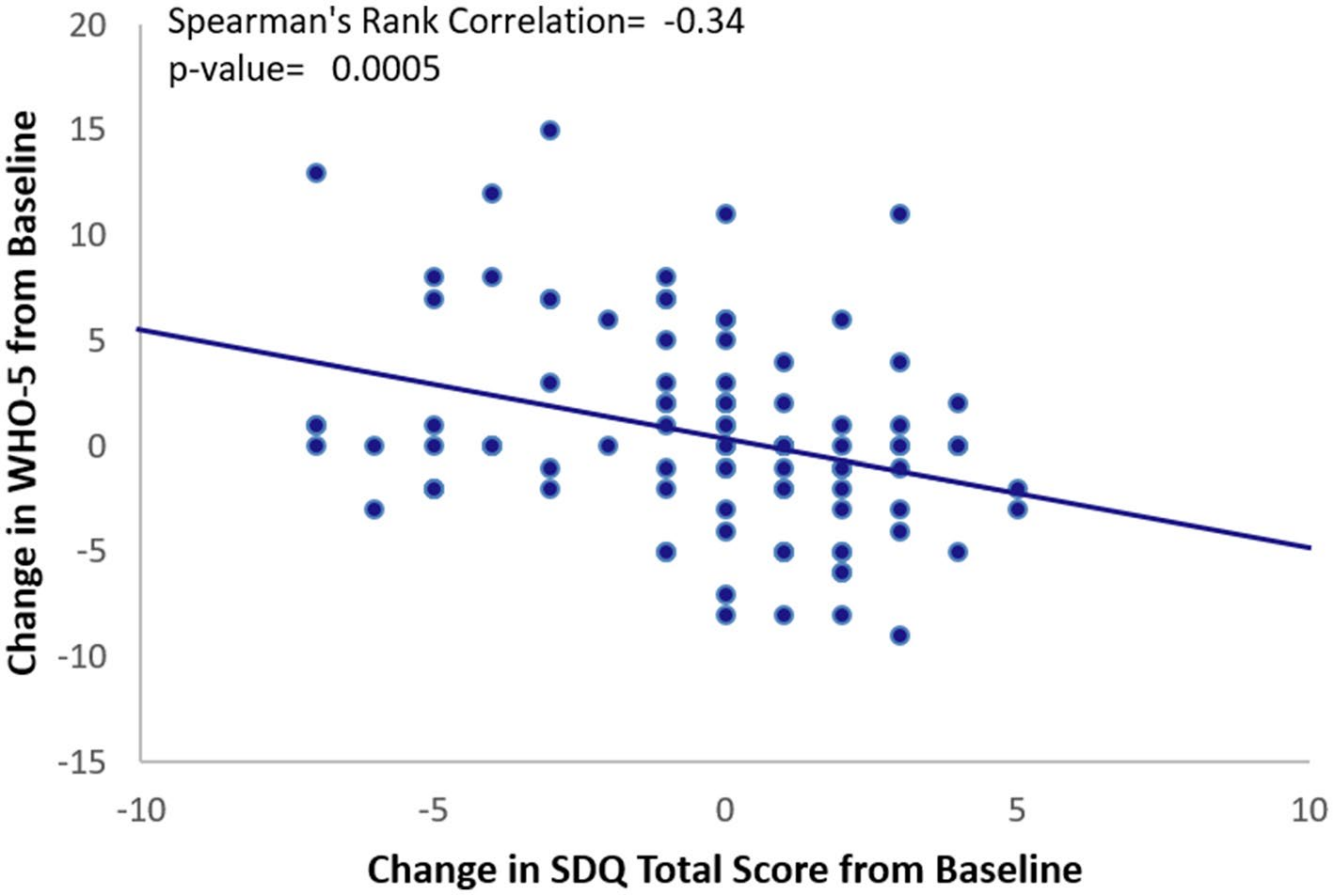
Spearman Rank Correlation between the change from baseline in WHO-5 Caregiver’s well-being and total SDQ score after 13 weeks of treatment with either PedPRM or placebo

There was a highly significant correlation between the improvement in caregiver’s quality of life (WHO-5) from baseline and the improvement (decrease) in total SDQ score (Spearman’s rank correlation − 0.34 p = 0.0005) (Fig. 3) but not with the SND reported changes in child sleep variables (TST, SL and duration of uninterrupted sleep).

### Safety

Safety data was detailed elsewhere (Gringras et al. 2017). In brief, PedPRM was generally safe; in the double-blind phase, as expected, somnolence was more commonly reported with PedPRM (28.3%) than placebo (10.8%).

## Discussion

The results presented here show that PedPRM treatment mainly improved externalizing behaviors (hyperactivity-inattention and conduct) in children and adolescents with ASD or NGD compared to placebo. In terms of clinical relevance, 53.7% of patients in the PedPRM group improved by 1 or more units on externalizing behavior score compared to 27.7% in the placebo group (Odds ratio 3.0; p = 0.008). Internalizing behaviors were not affected in the total population. Overall, 47% of subjects had a reduction of 1 or more in total SDQ score in the PedPRM group, compared to 27.1% in the placebo group (p = 0.035). Furthermore, when taking into account only those children and adolescents displaying abnormal hyperactivity/inattention scores at baseline, the total SDQ improved significantly in the PedPRM group compared to placebo. In the subpopulation with abnormal conduct scores, conduct improved significantly in the PedPRM-treated group compared to placebo.

The relevance of the improvement in these behavioral attributes to the improvement in sleep in the study population was further demonstrated by the significant correlations between the changes in TST and duration of uninterrupted sleep with the changes in total SDQ and less so for SL. The impact of sleep duration on behavior can thus be explained, at least in part, by the improvement in duration of uninterrupted sleep rather than the improvement in sleep latency. This is of note because the prolonged release formulation which releases melatonin throughout the night appears to be effective in improving both sleep onset and sleep maintenance whereas immediate release melatonin formulations are reportedly as effective in sleep induction but less so with sleep maintenance.

Previous studies suggested that healthy sleep has daytime benefits in TD children (Gordon 2000), in those with developmental disorders (Wasdell et al. 2008) and in children with ASD (Minde et al. 1994; Rossignol and Frye 2011; Wright et al. 2011). Moderate reduction of 41 min of the sleep period resulted in significant impairments in neurobehavioral functioning (Sadeh et al. 2003). Furthermore, a cumulative extension of sleep duration of 27.36 min was reportedly associated with detectable improvement in emotional lability and restless-impulsive behavior scores of children in school and a significant reduction in reported daytime sleepiness, whereas a cumulative restriction of sleep of 54.04 min was associated with detectable deterioration on such measures (Gruber et al. 2012). On average, children with ASD tend to sleep 32.8 min less per night and take almost 11 min longer to fall asleep than their TD peers (Elrod and Hood 2015); such sleep deficits most likely affect their daytime behavior, mental state and school performance.

In our study, children and adolescents with ASD or NGD slept on average 57.5 min longer at night with PedPRM compared to 9.14 min with placebo and their sleep latency decreased by 39.6 min on average with PedPRM compared to 12.5 min with placebo. The subsequent improvements in behavioral aspects reported here support the role of poor sleep in worsening behavioral problems, specifically hyperactivity/inattention. Importantly, the drug was effective in improving sleep variables (Gringras et al. 2017) as well as behavioral challenges in subjects with co-occurring ADHD as in those without an ADHD diagnosis. Giving a child the opportunity to sleep longer at night could reverse some of these behavioral consequences. These findings provide evidence of causality between the sleep problem and exacerbation of the externalizing behavior abnormality.

This study does not provide conclusive evidence as to changes in internalizing behavior when treating the sleep disorder. A limitation in our study was that due to variability in age and communication abilities, the evaluations were made by caregivers rather than by child reports and could not fully capture changes in internalizing behavior. A recent study, comparing self- and parent-reports in verbally fluent adolescents with ASD, highlighted the importance of considering adolescent-reports of negative peer experience, such as bullying, rather than relying exclusively on parent reports (Adams et al. 2014). Another possibility is that the internalizing behaviors are a cause rather than a result of the sleep problem. However, in our study those with abnormal peer relationship at baseline did improve with PedPRM treatment but not placebo, suggested that insufficient sleep does exacerbate these behaviors as well. Of note, there were less subjects in the study with abnormal internalizing behaviors than externalizing behaviors in the first place, and improvement is in general more difficult to demonstrate in the absence of rigorous baseline pathology.

Caregivers benefitted from their children’s treatment with PedPRM in improved quality of life in a statistically significant (p = 0.01) and clinically relevant manner (Effect Size 0.52) compared to treatment with placebo. As noted previously (Gringras et al. 2017), after 13 weeks of double-blind treatment, caregivers were more satisfied with their child’s sleep pattern in the PedPRM group compared to placebo. It is pertinent to ask whether the improvement in parents’ quality of life subsequent to the improvement in child’s sleep might provide a “positive bias” on the parent-completed rating scales of patient behavior or influence parenting and therefore behavior of children. Yet, the change in caregivers’ quality of life seems strongly related to the improvements in daytime behavior (total SDQ) rather than to the improvements in child’s sleep (TST, SL, or longest duration of uninterrupted sleep). Of note, caregivers’ quality of life had already improved significantly with the PedPRM treated compared to the placebo treated groups after 3 weeks treatment; by that time the hyperactivity/inattention scores also improved significantly in the PedPRM compared to the placebo treated groups. Furthermore, caregivers’ own sleep did not improve significantly with PedPRM compared to placebo and improved further only months later in the study (Maras et al. 2018). These findings could be interpreted as improvement of wellbeing of parents is mediated by improvement of daily behavior in the child and not directly by sleep improvement in the child or parent. It is therefore most likely that the improvement in sleep in the subjects led to the improvement in behavior and that this improvement is more valuable to the caregivers’ quality of life than the improvement in child sleep per se. These effects might benefit both the caregivers and the children, making caregivers more able to cope and potentially reducing stress in the parent–child relationship.

So far, two placebo controlled studies have looked into the effects of melatonin on behavioral aspects in children with neurodevelopmental disorders and insomnia. Only one of them has reported significant effects of melatonin compared to placebo on behavioral aspects of children with ASD and insomnia (Wright et al. 2011). In that study, children with ASD and sleep problems who were not amenable to behavioral management strategies received immediate release melatonin (mean dose 7 mg; range 2–10 mg) and placebo for 3 months in a crossover design. There was a significant improvement in overall daytime behavior with the immediate release melatonin compared with placebo as measured by the Developmental Behavior Checklist (DBC) (Wright et al. 2011). Whereas a change in total DBC score reportedly concurs with a change in total SDQ (Rice et al. 2018), no differences in treatment effects in any of the five attributes of the DBC checklist (disruptive behavior, self-absorbed, communication disturbance, anxiety and social relating difficulties) were observed (Wright et al. 2011) despite the higher doses used there compared to our study (prolonged release formulation, mean dose 3.8 mg; range 2–5 mg). In another study with immediate release melatonin, with similar design to the present study, child behavior and family functioning outcomes tended to favor melatonin but were not significant. These differences across studies may thus be related to its release formulation (immediate vs. prolonged release), and the lack of significant effects in previous studies could be due to the little additional sleep gained on immediate release melatonin (Gringras et al. 2012).

A limitation in our study was that the effects of the higher dose (10 mg) of PedPRM treatment on behavior was not explored. The 10 mg dose was allowed only in the later open label follow up phase to further improve sleep in subjects who did not respond to the lower doses (Maras et al. 2018). Unfortunately, no behavioral data were collected at the late phases. Future trials should consider measuring the effects of higher PedPRM doses on child behavior.

Another limitation is that the study was powered to detect differences in the effects on sleep and not on the behavioral endpoints, so a lack of significance could be due to a lack of power. Furthermore, because most of the endpoints were exploratory, no correction for multiple comparisons was performed, which could in principle lead to type 1 errors. Nevertheless, the reliability of the findings lies in the consistency in effects (specifically hyperactivity/inattention) across time points and subpopulations in the study and the significant correlations between effects demonstrated with independent tools.

It has been suggested that children and adults with higher-level cognitive functioning are more affected by reduced sleep than those functioning at lower cognitive levels (Gomez et al. 2011). The scope of our study did not allow us to investigate intellectual and school performance of the patients in this multi-center trial because we could not ascertain consistency of informants. Future trials should consider measuring the effects of PedPRM treatment on school performance of the children.

The importance of the PedPRM induced alleviation of externalizing behavioral difficulties, even more so in subjects with abnormal hyperactivity/inattention at baseline, may extend beyond the behavioral aspect alone. This is because presence of ADHD symptoms in the context of ASD could have a variety of effects on cognition, adaptive/maladaptive behavior (Murray 2010), verbal working memory (Yerys et al. 2009) motor problems (Murray 2010), resulting in a stronger severity of autistic symptoms (Sprenger et al. 2013) and more severe comorbid symptoms (Jang et al. 2013) than patients with ASD only. According to Posey and McDougle (2000) educational and behavioural interventions are unlikely to reach their optimal efficacy in children with ASD and ADHD symptoms without adequate control of symptoms of inattention and hyperactivity. The improvement in externalizing behaviour with PedPRM is thus key to successful intervention in this population. Future longitudinal studies should address these long-term effects on symptom trajectories in children and adolescents with ASD with and without co-occurring ADHD.

Overall, PedPRM treatment of insomnia in children with ASD with and without co-occurring ADHD significantly alleviates insomnia-related difficulties in externalizing behavior, and subsequently improves caregivers’ quality of life.
